# Which Body Would You Like to Have? The Impact of Embodied Perspective on Body Perception and Body Evaluation in Immersive Virtual Reality

**DOI:** 10.3389/frobt.2020.00031

**Published:** 2020-03-18

**Authors:** Solène Neyret, Anna I. Bellido Rivas, Xavi Navarro, Mel Slater

**Affiliations:** ^1^Event Lab, Department of Clinical Psychology and Psychobiology, University of Barcelona, Barcelona, Spain; ^2^The Institute of Neurosciences, University of Barcelona, Barcelona, Spain

**Keywords:** body perception, immersive virtual reality, visual perspective, body image, body evaluation, eating disorders, body satisfaction

## Abstract

In this experiment, we aimed to measure the conscious internal representation of one's body appearance and allow the participants to compare this to their ideal body appearance and to their real body appearance. We created a virtual representation of the internal image participants had of their own body shape. We also created a virtual body corresponding to the internal representation they had of their ideal body shape, and we built another virtual body based on their real body measures. Participants saw the three different virtual bodies from an embodied first-person perspective and from a third-person perspective and had to evaluate the appearance of those virtual bodies. We observed that female participants evaluated their real body as more attractive when they saw it from a third-person perspective, and that their level of body dissatisfaction was lower after the experimental procedure. We believe that third-person perspective allowed female participants to perceive their real body shape without applying the negative prior beliefs usually associated to the “self”, and that this resulted in a more positive evaluation of their body shape. We speculate that this method could be applied with patients suffering from eating disorders, by making their body perception more realistic and therefore improve their body satisfaction.

## Introduction

Defining the boundaries of a person necessarily requires the consideration of their body (Tsakiris, 2016). The body is one element of the individual that is clearly spatially demarcated. Proprioception and visual information combine to create an internal representation of one's body (Gallagher, 1986). This representation is involved in motor control and in the construction of a body image (Bermúdez et al., 1995). However, what may be perceived as the body of a person by him- or herself might not correspond to an objectively accurate description of their body. We know that body representation of oneself is not necessarily tuned to the real spatial boundaries of the body and can result in an overestimation of body parts (Longo and Haggard, 2010, 2012; Thaler et al., 2018a).

It is known that there are different levels of body representation that are informed by different modalities, and that determine how the own body and its size are perceived (de Vignemont, 2010, 2011). Implicit representations are thought to be informed by proprioception, somatosensation, and interoception, whereas explicit representations are thought to be constructed based on visual perception of the body and cognitive-affective factors (Thaler et al., 2018c). It is also known, that the visual information that one receives when observing the body of conspecifics can be integrated to their own body representation (Calvo-Merino et al., 2005). Body representation includes some visual references from both self-perception and perception of others. It has been shown that other bodies perceived in one's surrounding environment influence the internal reference of what is perceived as average body but also play an essential role in self-body size evaluation via social comparison (Cattarin et al., 2000). Socio-cultural surrounding therefore have a major role in body perception, body representation, and body evaluation. Humans are normally unable to perceive their own body from a third person perspective, except when seeing a picture or a video of themselves. The third-person perspective provides an holistic view of one's body, it is the same perspective one has of other people's bodies. This perspective is therefore thought to be involved in self-other comparisons (Thaler et al., 2019). It has been shown that humans construct an internal visualization of their body, based on the visual information perceived in other bodies, through visual imagery (Decety, 1996; Naito et al., 2002). It has also been shown that one's body shape has a direct impact on the perception of self and other's bodies (Thaler et al., 2016, 2017), and that virtual reality is a particularly efficient tool for studying body perception of self and others (Piryankova et al., 2014a,b; Nimcharoen et al., 2019). In the experiment reported in this paper, we explore to what extent prior beliefs about the self and about others influence body evaluation.

There are various definitions of body representation and how it is related to a conceptual sense of self. Minimal self and the sense of agency define the sense of “mineness” and “ipseity”, being a pre-reflective proprioceptive, ecological sense of self that contributes to the basic differentiation between self and non-self (Gallagher, 2000). Self-representation is developed as a meaningful structure at the level of our bodily movements through space, in the basic interaction between an organism and its environment (Neisser, 2008). One critical element appearing in all the different definitions is the conscious aspect of being a self through the subjective experience of having a body (Metzinger, 2014). One common differentiation found in the literature is the distinction between “body image” and “body schema” (Gallagher, 1986, 2000, 2005; Kammers et al., 2009). The body image is defined as a relatively constant, conscious representation of one's body including conceptual beliefs about the self, whereas the body schema is defined as an unconscious and very plastic representation. De Vignemont proposed that the main distinctions between body schema and body image are in the temporality of the representation (long term vs. short term), its availability to consciousness and its functional role (action vs. perception) (de Vignemont, 2010). In the experiment described here, we use the term “body image” in the sense of the conscious, long term, pre-existing representation involved in body perception of oneself. We will observe how body image is closely related to prior beliefs about the self (Longo et al., 2009; Perez-Marcos et al., 2018) and how body perception and body evaluation directly depend on these prior beliefs.

Since we used a metric method for the body estimation task, we expected inaccuracy, distortion and overestimation of the size of the body parts, as has been reported in the literature (Longo and Haggard, 2010, 2012; Thaler et al., 2018b). Our aim was to provide a means whereby participants might see how inaccurate their internal (implicit) perception of their body is. We showed them a virtual body recreated from their body size estimation (based on the combination of their visual and somatosensory body representation), directly standing next to an avatar recreated from their real body measures. We expected that their body satisfaction would be improved after seeing how inaccurate their internal body representation was. It has been shown that the degree of distortion in body representation (mainly overestimation of certain critical body parts) is correlated with body dissatisfaction in anorexia nervosa (Garner and Garfinkel, 1981). This experiment was aimed at providing evidence toward a method that could then be used in patients with eating disorders, for whom body dissatisfaction is very high and body representation is highly distorted.

## Materials and Methods

### Equipment

#### Head-Mounted Display

**Figure 1:**
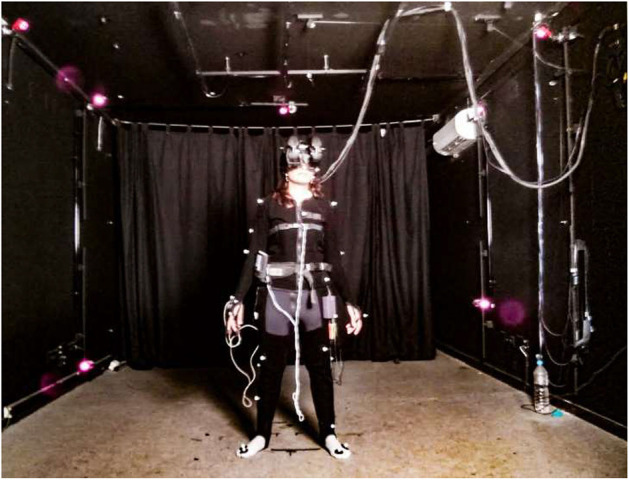
A participant wearing the Optitrack body suit and the nVision SX111 HMD.

We used the nVision SX111 head-mounted-display (HMD) made by NVIS (Figure 1). It displays a 3D scene in stereo with a horizontal field of view of 102 degrees and vertical field of view of 64 degrees by sending left-eye and right-eye images to left and right screens. Its weight is 1.3 Kg. An Intersense Tracker mounted on top tracks the head movements of the participant with 6 degrees of freedom, and updates the images displayed accordingly, allowing participants to experience visuo-motor correlation between their head movements and the displayed virtual environment. From the point of view of the participant, it is equivalent to using their head gaze normally to look around a scene.

#### Optitrack Motion Capture System

The Optitrack motion capture system is designed to support real-time whole-body tracking of a participant (http://www.optitrack.com). It includes a Velcro suit with 28 retroreflective markers (Figure 1), which are tracked by 12 infrared cameras. It also includes small sets of 3 markers used to track the hand position of the participants.

#### Tactile Feedback

**Figure 2:**
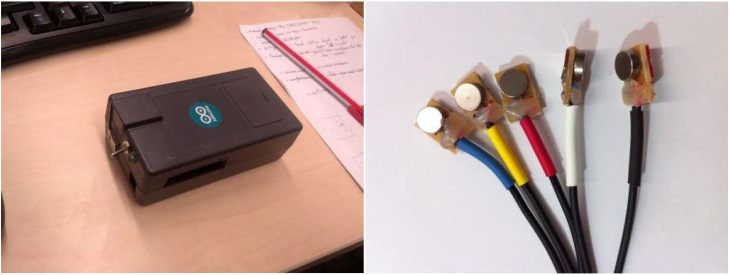
Arduino board **(Left)** and vibrators **(Right)**.

We provided participants with tactile feedback by using an Arduino board that controlled four small vibrator devices (Figure 2). Four vibrators were placed on the participant, one on each leg in the middle point between the knee and the foot, and one on each hand. The Arduino board operates via Zigbee. These vibrators were used to give correspondingly located visuo-tactile feedback to the participants when virtual objects entered in contact with their virtual arm or virtual legs (see experimental procedure).

### Participants and Recruitment

We recruited 11 males and 12 females from the campus of the University of Barcelona. Participants were between 18 and 38 years old, the mean ± SD age of the sample was 24.8 ± 5.64. Recruitment was carried out by announcement through leaflets around the campus and by emailing our data-base for participation in experimental studies, all according to the rules of the Data Protection Office of the University of Barcelona. We had to exclude one male and three females because their scores were above the threshold on the clinical questionnaires (see below). All participants had normal or corrected-to-normal vision and were screened for contra-indications for VR through pre-experimental forms (e.g., epilepsy, recent alcohol intake, psychoactive drugs treatment). The total compensation was ten euros. The study was approved by the *Comissió de Bioètica* of the University of Barcelona. Participants were asked to attend the Virtual Reality laboratory on two occasions. They received the general information about the experimental procedure and signed the informed consent. We did not use any questionnaire related to simulator sickness given that the participants did not experience translation of their viewpoint through the virtual environment. We created the effect of embodiment through visuo-tactile correlation and only head movements were allowed during the course of the experiment. Participants were told that the aim of the experiment would be explained after the end of the second experimental session and therefore they remained naïve about the goal of the experiment until the end of the procedure. No side effects of the virtual reality were reported. This was measured using a follow up procedure validated through the *Comissió de Bioètica* and the data protection office of the University of Barcelona. The questionnaire was sent to all the participants by email 1 month after they had completed the experimental procedure.

### Clinical Questionnaires

Participants completed two psychological tests evaluating Body satisfaction and Eating Disorders symptomatology (BSQ-34 and EDI-2); high scores on one of these questionnaires, showing a pathological tendency to Eating Disorders, was an exclusion criterion. We isolated the results of two subscales of the EDI-2 that were the most relevant for the purpose of our study: Drive for thinness (DT) and Body dissatisfaction (BD). The results of both questionnaires are reported in Tables S3–S5 (Supplementary Material).

#### EDI-2: Eating Disorders Inventory

The EDI-2 is a self-report questionnaire composed of 91 items rated from 0 to 5 (0: Never, 5: Always). It was developed in order to evaluate the symptoms associated to eating disorders. It includes 11 subscales: Drive for Thinness, Bulimia, Body Dissatisfaction, Ineffectiveness, Perfectionism, Interpersonal Distrust, Interoceptive Awareness, Maturity fears, Ascetism, Impulse Regulation, Social Insecurity (Garner et al., 1983). It has been adapted to Spanish and has shown good internal validity (García-García et al., 2003). Here we used the Spanish version.

#### BSQ-34: Body Shape Questionnaire

The BSQ-34 is a self-report questionnaire of 34 items measuring how the individual has been feeling about their body appearance over the past 4 weeks. It was developed and validated in clinical and non-clinical populations (Cooper et al., 1987). All the items are rated on a Likert scale ranging from 1 to 6 (1: Never, 6: Always). In this experiment we used the adapted Spanish version (Raich et al., 1996).

### Experimental Procedure

In the experiment reported here we aimed to use virtual human bodies (avatars) for self-representation, thereby allowing the possibility for participants to perceive the difference between the internal model they have of their own body and their actual body shape. We used a new method that could be defined as an “interactive metric” method, given that participants had to estimate the size of their body parts using the length of a virtual tube that they were holding between their hands. We also aimed to test whether the evaluation of a virtual body representing the self would vary depending on the perspective from which it was perceived. Participants were able to see this representation of their body from a third person perspective (as if it was the body of someone else), and from an embodied first-person perspective (by looking down to their body and by perceiving it in a mirror).

In the first session of the experiment we used an interactive metric method enabling us to measure participants' representations of their own body (body image) and to re-create an avatar corresponding to these measures (see description of the method in “*Body shape estimation*”). In addition to that, we asked participants to create an estimation of their *ideal* body shape and we created another avatar corresponding to this. In order to compare those bodies generated from subjective implicit representation, the supposed actual one and the ideal one, we also measured the real body shape of the participant and generated a third avatar corresponding closely to their real body appearance. The avatar based on the real body measures obtained through the Optitrack device (Figure 1) was called the Real Body (RB), the second avatar based on the estimations participants gave of their subjective body representation, was called Body Image (BI) and the third avatar based on the estimations participants gave of their ideal body shape, was called Ideal Body (IB).

In the second session of the experiment (1 week after the first session) participants saw the created avatars without knowing which one corresponded to the Real Body, Ideal Body, or Body Image. We asked the participants to evaluate the appearance of the three virtual bodies. There were two conditions for this subjective evaluation: one in first person perspective (in which the participants were embodied in the virtual body), the other in third person perspective. We expected that prior beliefs about the self would negatively affect the way participants would perceive and evaluate their avatars in first person perspective.

#### Session 1

Participants arrived at the laboratory and read the information corresponding to the first phase of the experiment. After signing the corresponding consent form, they first filled in a demographic questionnaire (see Tables S1, S2), and then filled in the two clinical questionnaires (EDI-2 and BSQ, see description in the Clinical Questionnaire section).

##### Object estimation task

After completing the questionnaires and before putting on the HMD, the participants were asked to inspect a chair from the laboratory. We instructed them not to pay attention to anything else but its proportions without touching it (we did not want them to remember the chair proportions in relation to their body). Once in the virtual environment, the participant had to estimate the width of the seat of the chair and the height of its back using what we will refer as the “virtual cylinder technique.” A virtual cylinder appeared between the participant's hands inside the virtual environment. It was generated based on the hands' positions tracked inside the virtual environment by a set of three retro-reflective markers from the Optitrack body tracking system (described in Equipment). Participants had to modulate the cylinder length by bringing their hands together or moving them away from each other (as if they were holding a flexible cylinder between their hands). Once the participant thought that the cylinder length corresponded to the measure they were asked to estimate, they indicated this fact verbally to the experimenter. If the distance estimated was correct the cylinder turned green, if the distance estimated was wrong the cylinder turned red (where the allowed error was 5 cm). They continued this estimation until they were correct. The virtual cylinder technique was specially created for this research. We designed this technique in order to measure the implicit perception participants had of the size of their body parts. We did not want participants to have a reference object with fixed size in order to ensure that the estimation would not be biased by external references. Our aim was to design a specific “interactive metric” method. Having participants using the length of a virtual tube that they were holding between their hands appeared to us as a natural way to estimate body parts sizes. We surmised that using their hands would allow participants to use (only) their own proprioceptive information as an inner reference for the estimation task. We added a training phase (estimating the size of the chair and estimating the body parts of a gender matched avatar) in order for participants to become familiar with the method.

##### Virtual reality scenario

**Figure 3:**
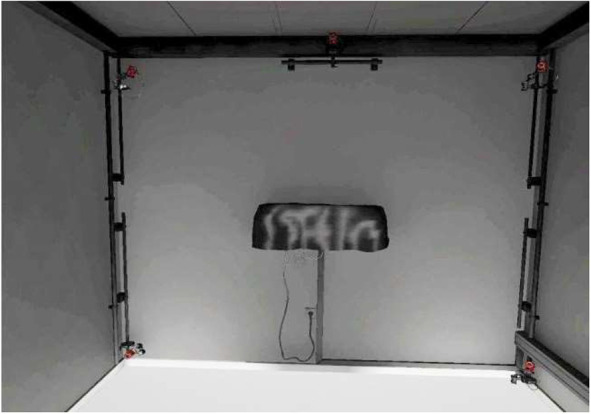
Model of the laboratory displayed in the virtual environment.

The virtual scene consisted of the reproduction of the real virtual reality laboratory in which the experiment took place (Figure 3). We decided to use this scenario since the only important aspect of the VR experience were the bodies, so we put participants in a particularly familiar environment (representing the actual space in which they were located) in order not to detract from perception of the bodies.

##### Training phase

Participants estimated their body parts using the cylinder method described above. However, pilot studies found that participants had difficulties estimating the size of each body part to which we referred. Therefore, we included a training task in which the participants were asked to estimate the body parts of a gender matched avatar standing in front of them in the virtual environment. For each part of the body that had to be estimated, participants were able to navigate inside the virtual environment, in order to inspect the avatar closely, and had as much time as they wanted. Once participants considered they had memorized the size of the body part they were asked to estimate, they would indicate it to the experimenter and the avatar in front of them disappeared. When the estimated size was wrong (within 5 cm), the gender matched avatar was shown again, and participants repeated the estimation task. The training phase was over when the participants managed to estimate accurately all the body parts of the gender matched avatar.

The set of body parts estimated depended on the gender of the participant. Indeed, we noticed during the pilot phase of the study that males and females do not focus on the same parts of their body to create a mental representation of their body shape. The size of the biceps was more critical in males and women gave more attention to the width of their waist. The final measures required by gender were:

- Male measures: hip width, hip depth, abdominal width, chest width, chest depth, thigh width, biceps width, neck width, distance between shoulders.
- Female measures: hip width, hip depth, waist width, waist depth, chest width, chest depth, thigh width, neck width, distance between shoulders.

##### Body shape estimation

**Figure 4:**
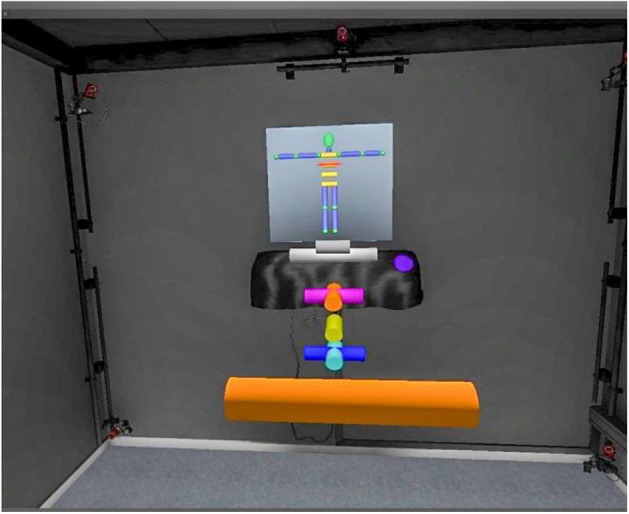
The body structure constructed by the participant (colored cylinders placed behind the orange cylinder) and the schematic picture on the wall (the part that needed to be estimated was highlighted in red on the schematic picture, here “chest width”). The orange cylinder here is the one used for the estimation, the other cylinders are already placed in space corresponding to the other body parts estimated by the participants.

After the training phase, the task of “Body shape estimation” was performed using the same virtual cylinder technique. However, this time participants had to estimate their own body shape, and no “body representation" was displayed. During this phase there was no feedback about the accuracy of the measures given. The set of body measures required were the same as those provided during the training phase. Participants were able to see all the tubes collocated in space and were able to visualize the whole “body structure” they were constructing (Figure 4). This method was inspired from the paper by Myers et al. (1992) in which three projected bands of light represent the size of the chest, waist, and hips of the participant.

Participants could correct the size of each cylinder as many times as they wanted until the body structure they constructed appeared correct to them. As there were some measures of depth, they were also able to navigate in the virtual environment in order to see the body structure in 3D. After estimating the measures of their real body, the participants were asked to construct their ideal body structure using the same method, where we had instructed them to imagine “the body they would like to have ideally” (height was not modifiable). The data gathered after this second part was used to generate two avatars per participant: one representing their Body Image (BI) and the other representing their Ideal Body (IB). In the case of this experiment, the Body Image was always estimated first and before the Ideal Body, this is a possible limitation of our study and we will consider order randomization for the “body shape estimation task” in future work.

##### Method used for generating real body avatars

**Figure 5:**
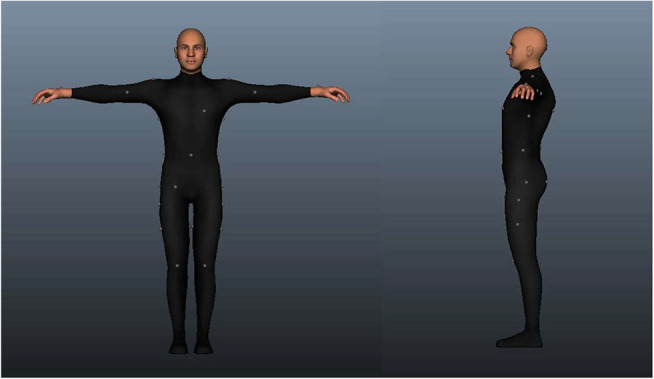
Position of the 32 markers on the body of the participant, 1 marker on the top of the head, 4 markers on each arm, 8 markers on the torso, 5 markers on the hips and buttocks, 4 markers on each leg, 1 marker on each foot.

At the end of this first session, participants were asked to put on the Optitrack suit (Figure 1) and we placed 32 markers on their body (Figure 5). The positional data extracted from those markers was used to generate an avatar representing their Real Body (RB). The RB avatars were generated using the 3D animation software Autodesk Maya 2012 (https://www.autodesk.com/products/maya). We created a program that allowed the adaptation of a set of body measures to a pre-constructed avatar. The problem to solve was to be able to adapt an avatar's body, be it male or female, to a set of body size constraints. The adaptation required needed to be coherent throughout the body, meaning that a very fat belly implied a consequent modification such as a double chin and wide cheeks. This was approached by using a pre-constructed avatar for each gender with five full avatar shapes (blendshapes): “thin”, “body builder”, “obese”, “beer belly”, and “straight body.” We wrote a script in MEL (Maya Embedded Language) to optimize the combination of these shapes in order to fit with the body part sizes. The resulting body was the closest to the set of distances, although it did not match those precisely. Therefore, a posterior process altered each body part slightly in order to address the final adaptation needs. After all the computations, the final avatar was a full-body deformation with a human and coherent appearance. Each avatar was generated in less than 10 min, including the introduction of the input data, the internal calculations and the final adaptation of the poses, accessories (virtual headset) and animations.

#### Session 2

**Figure 6:**
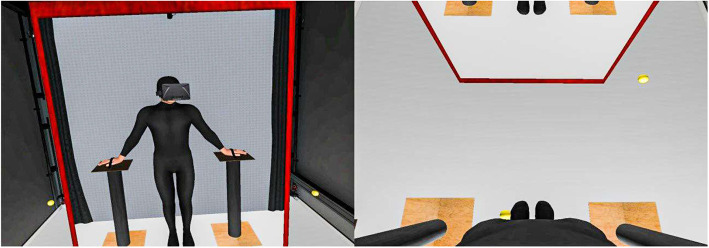
Avatar seen in first-person perspective, on the left, front view of the avatar reflected in the mirror and yellow balls moving toward the legs to create visuo-tactile feedback. On the right, view of the virtual body when the participant looks down.

**Figure 7:**
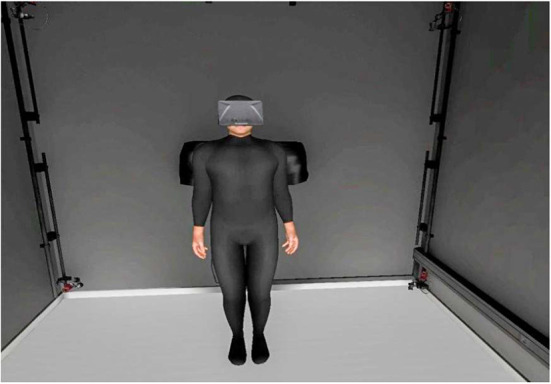
Avatar seen in third-person perspective, from the point of view of the participant.

During the second session (1 week later), participants were able to see the virtual representation of their three Avatars (inside the virtual environment) from two different perspectives: first person perspective (1PP), and third person perspective (3PP), as shown in Figures 6, 7.

In total, participants saw six virtual bodies that they had to evaluate. These were three different avatars representing respectively their Ideal Body, Real Body, and Body Image, with each of the avatars seen once in first person perspective and once in third person perspective. The avatars were presented randomly with respect to the type of body and to the perspective, always alternating between first person perspective and third person perspective. All avatars were dressed in a black body suit (similar to the Optitrack body suit the participants were wearing) and had a virtual model of the Oculus rift on their head (similar to the nVis HMD in appearance). All avatars were consistent with the participant's gender. At the end of the second session, the three avatars corresponding to the BI, IB, and RB of each participant were presented simultaneously in 3PP. Participants were asked to guess “which of those was their real body”, and to choose “which of those bodies they would like to have.”

In the 1PP condition (Figure 6), we aimed to induce body ownership over the virtual body in order for the participants to apply to that body the prior beliefs associated to themselves. We used visuo-motor correlation and synchronized head movements between the participant and the avatar. There was a virtual mirror in front of the participants in which they could see themselves moving their head. Each participant's hands were placed on two modules covered with a rough brown material. Their real body posture corresponded to the posture of the virtual body (co-located body) and the two modules were reproduced in the virtual environment (haptic feedback). Participants could see their virtual hands and legs by looking down toward themselves. We gave special attention to reproducing exactly the appearance of the rough brown texture covering the two modules to enhance the illusion of body ownership toward the virtual hands through visuo-tactile feedback. In addition, four small yellow balls moved back and forth in the virtual environment touching the participants' hands and legs in a continuous movement (see Figure 6). The vibrators (described in Equipment) were activated synchronously when the balls touched the participant's virtual body in order to reinforce embodiment over the virtual body (Petkova and Ehrsson, 2008; Bourdin et al., 2017).

In the third person perspective condition (Figure 7), we did not require body ownership over the virtual body, the goal was for the participants to perceive the virtual body that was displayed in front of them as if it were the body of someone else. In this case, the head movements of the avatar were independent of the participant's head movements, the avatar had a different posture and performed a pre-recorded animation, so that there was no motor correspondence between the participant's movements and the avatar's movements. Participants had no visual feedback of their body when they looked down.

The subjective evaluation of the avatars was based on a verbal questionnaire in which participants had to judge several aspects of the appearance of each avatar while they were seeing it, inside VR. The order of the questions was randomized for each “evaluation”, but the Body Ownership questions were always first, as we did not want the participant to confuse those with questions related to the physical appearance of the avatar. This way, the two blocks of questions were more clearly differentiated. The participants rated their answers on a seven-point Likert scale where 1 meant “not at all” and 7 meant “very much.”

After the questionnaires had been completed, we provided each participant with personalized feedback, telling them which avatar corresponded to their real body, ideal body, body image. We were interested to see whether the participants would perceive their real body shape from a new perspective based on this information. Hence, immediately after this, participants completed again the EDI-2 questionnaire in order to measure if this experience would have an immediate effect on self-evaluation.

## Results

### Body Ownership

**Figure 8:**
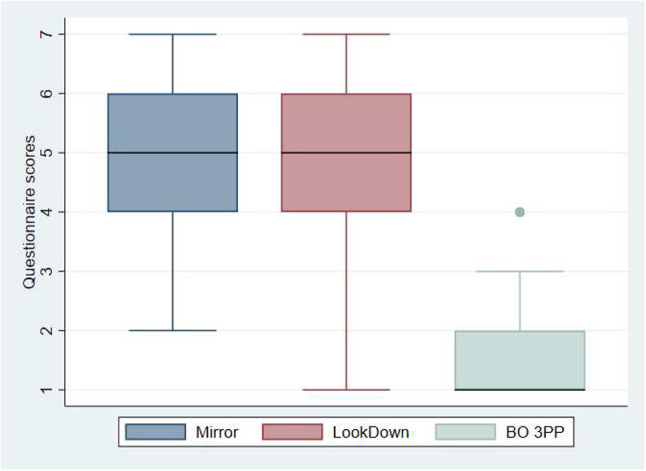
Box plot presenting the level of body ownership depending on the perspective. The horizontal thick lines indicate the value of the medians, the boxes are the interquartile ranges (IQR), the whiskers extend from max (median - 1.5*IQR, smallest value) to min (median + 1.5*IQR, largest value). Outliers are shown individually as separated dots. Mirror and LookDown correspond to the level of body ownership for all the avatars when presented in 1PP. BO 3PP corresponds to the level of body ownership for all the avatars when presented in 3PP.

In the first-person perspective condition, participants were embodied in the virtual body, and there was visuo-tactile stimulation on their legs and hands (Figure 6). We assessed the level of body ownership participants were experiencing, each time the virtual bodies were presented in 1PP. We measured the level of body ownership when the virtual bodies were presented in 3PP as well, to make sure that the 1PP was triggering the effect we wanted. Results show that the level of body ownership was almost not existent in 3PP and very high in 1PP (Figure 8). The questionnaire was the following:

**Table:** 

**Body ownership questionnaire 1PP:Likert scale, 1: not at all, 7: very much**	**Variable name**
¿Hasta qué punto sientes que el cuerpo que ves en el espejo es tuyo (sin tener en cuenta el nivel de semejanza física entre este cuerpo y el tuyo)? ¿*To what extent do you feel the body you see in the mirror is yours (not considering physical resemblance)?*	Mirror
¿Cuándo miras hacia abajo, hasta qué punto sientes que el cuerpo que ves es tuyo? *When you look down how much do you feel that the body you see is yours?*	LookDown

**Table:** 

**Body ownership questionnaire 3PP:Likert scale, 1: not at all, 7: very much**	**Variable name**
¿Hasta qué punto sientes que el cuerpo en frente de ti es tuyo (sin tener en cuenta el nivel de semejanza física entre este cuerpo y el tuyo)? ¿*To what extent do you feel the body you see in front of you is yours (not considering physical resemblance)?*	BO 3PP

The illusion of body ownership was high (median = 5) in the first-person perspective condition (Mirror and LookDown) and low (median = 1) in the third-person perspective condition (BO 3PP). The evidence suggests that the visuo-tactile integration elicited on the hands and legs of our participants by the synchronization of the virtual balls entering in contact with their virtual body and the vibration felt on their real body, created a strong body ownership illusion on all the avatars presented in 1PP, independently of the avatar's appearance. On the contrary, there was no body ownership illusion over the avatars when presented in 3PP.

### Clinical Questionnaires

#### BSQ-34

The Body shape questionnaire (see Table S3), was completed after the first session (BSQ_PRE) and also after the second session (BSQ_POST). We observed that the female participants had more concern with their body shape than the male participants. Since we measured the same group of individuals at two different times, a paired *t*-test was adapted to test for significance in the difference observed. There was no significant difference between the BSQ score before and after the experimental procedure for females (*p* = 0.50), and neither for males (*p* = 0.96).

#### EDI-2

This questionnaire was used for the screening in order to make sure that the sample was formed only of psychologically healthy participants. Additionally, we wanted to compare the results before and after the experimental procedure to see if there would be any effect on any of the subscales. Here we report the results obtained for the subscales: Body dissatisfaction (BD) and Drive for thinness (DT). All the results are presented separately for males and females since there are critical differences between the two groups (see Tables S4, S5). “Pre” refers to the scores obtained before the experiment, “Post” refers to the scores obtained after seeing the six avatars in 1PP and 3PP, right after the second session of the experimental procedure. We observed a decrease in the scores of the “Drive for thinness” scale, in female participants. This effect is not observed in male participants. The DT scores measured before the experimental procedure are much higher for females than for males. We ran a paired *t*-test to see whether the difference of the DT scores before and after the VR session was significant, but it was not in females (*p* = 0.32) nor in males (*p* = 0.55). We ran a paired *t*-test and the difference in body dissatisfaction before and after the experiment was significant for females (*p* = 0.04) but not for males (*p* = 0.19). It seems that the experimental procedure of seeing the different avatars representing their body, resulted in a tendency to reduce body dissatisfaction in females. We believe the feedback given to the participants about the real shape of their body led them to get a better self-evaluation and less body dissatisfaction.

### Averaged Avatars

**Figure 9:**
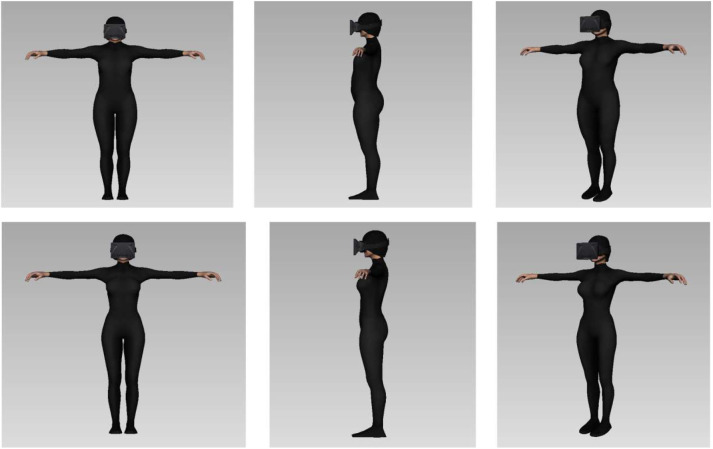
On the top, average shape of the body image avatars showing the mean (over)estimation females gave of their own body shape. Below, averaged shape of the ideal body avatars generated with the mean estimation females gave of their ideal body measures.

**Figure 10:**
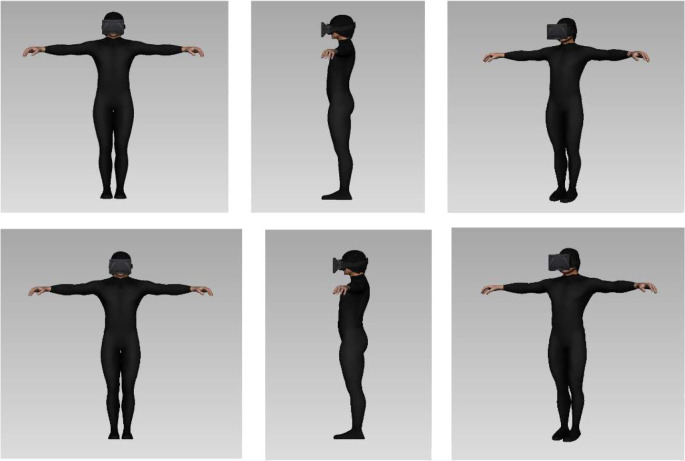
On the top, average shape of the body image avatars showing the mean estimation males gave of their own body shape. Below, average shape of the ideal body avatars showing the mean estimation males gave of their ideal body shape.

Here we present pictures corresponding to the avatars that were generated based on the estimations participants gave of their own body (BI) and ideal body (IB). We present four averaged avatars corresponding to the females' body image and ideal body (Figure 9), and to the males' body image and ideal body (Figure 10).

We can observe that the ideal body image is significantly thinner than the Body Image avatar for females. The critical body measures are the “waist width” and “hips width”, that are smaller in the ideal female averaged body. It is interesting to note that the ideal body shape we obtained corresponds to the “hour glass” shape reported in Simmons et al. (2004), see the Discussion for further development on those results.

### Subjective Evaluation of the Avatars

In the second part of the experiment the three avatars generated (Real, Ideal, and Body Image) were presented to the participants in 1PP or 3PP in a random order. Each avatar was perceived in both perspectives and we measured the subjective evaluation for each avatar in each condition.

**Table:** 

**Evaluation of the AvatarsLikert scale from 1 to 7, 1: not at all, 7: very much**	**Name of variable**
¿Cuán gordo crees que es este cuerpo? *How fat do you think this body is?*	Fat
¿Cuán delgado crees que es este cuerpo? *How thin do you think this body is?*	Thin
¿Cuánto te gustaría que tu cuerpo se pareciera a este? *How much would you like your body to resemble this one?*	LikeToResemble
¿Cuán atractivo te parece este cuerpo? *How attractive does this body look to you?*	Attractive

#### Results for female participants

**Figure 11:**
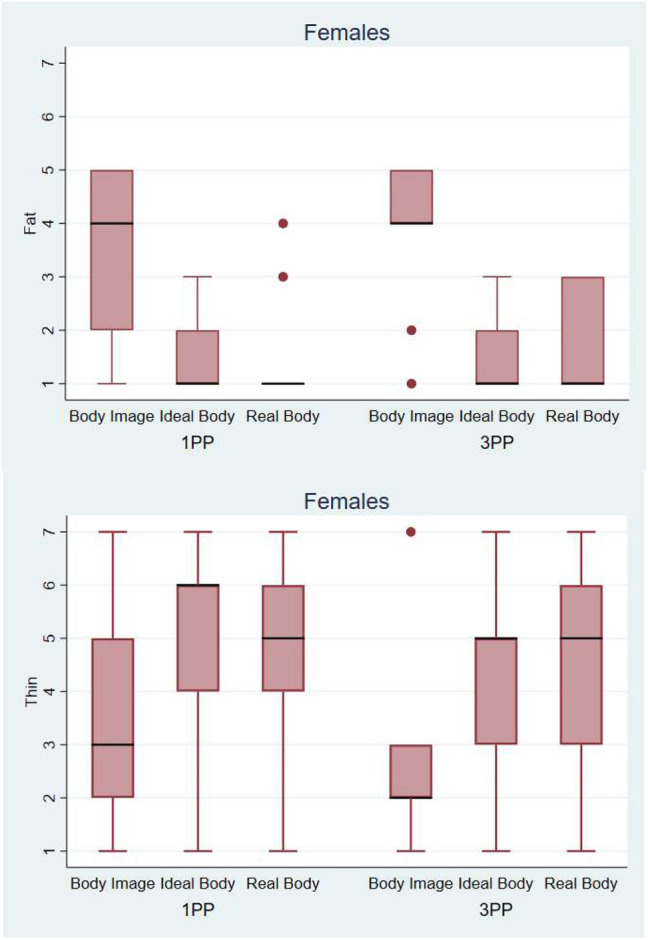
On the top Box plot presenting the scores obtained during the body evaluation phase. Answers to the question: *How fat do you think this body is*?—Below Box plot presenting the scores obtained during the body evaluation phase. Answers to the question: *How thin do you think this body is*? The horizontal thick lines indicate the value of the medians, the boxes are the interquartile ranges (IQR), the whiskers extend from max (median - 1.5*IQR, smallest value) to min (median + 1.5*IQR, largest value). Outliers are shown individually as separated dots.

In Figure 11 we can observe that the female participants evaluated the avatar corresponding to their Body Image as fatter (median = 4) than the avatars corresponding to their Real Body and their Ideal Body. The body image avatars were indeed “objectively” fatter than the real body avatars because female participants tended to overestimate the size of their own body (Figure 9). Logistic regression with fixed effects over Perspective and Body and random effects over the individuals shows that there was no interaction effect (*p* = 0.4) but strong main effects, with both Ideal Body and Real Body being evaluated as significantly thinner than the Body Image (*p* = 0.0005). For the question about the thinness of the avatar we observe the same effect as in the previous question, the Body Image avatar is perceived as significantly fatter than the other two avatars, from both 3PP and 1PP.

**Figure 12:**
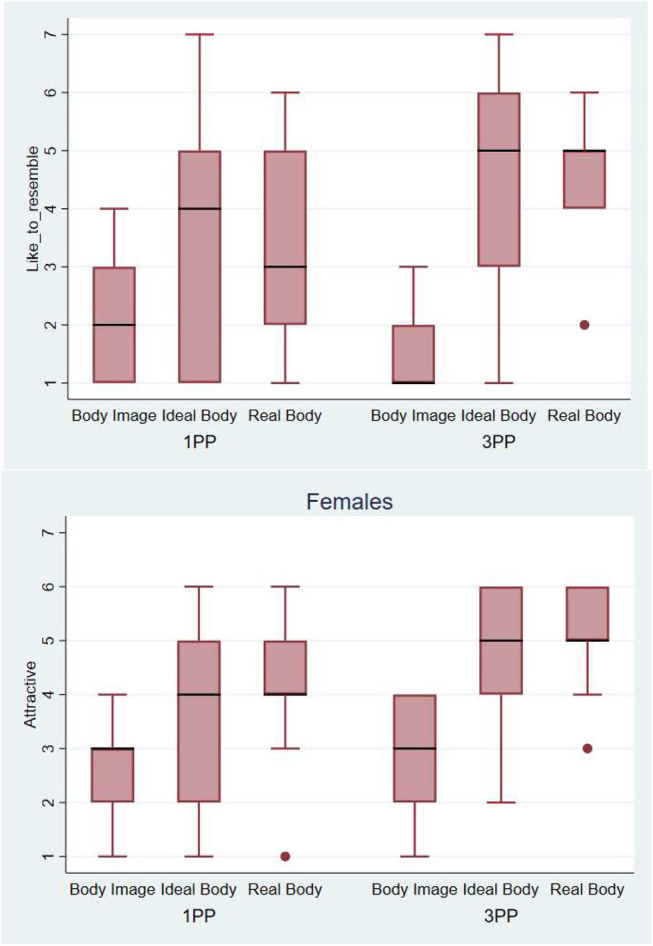
On the top, Box plot presenting the scores obtained during the body evaluation phase. Answers to the question: *How much would you like your body to resemble this one?*—Below, Box plot presenting the scores obtained during the evaluation phase. Answers to the question: *How attractive does this body look to you?* The horizontal thick lines indicate the value of the medians, the boxes are the interquartile ranges (IQR), the whiskers extend from max (median - 1.5*IQR, smallest value) to min (median + 1.5*IQR, largest value). Outliers are shown individually as separated dots.

Figure 12 shows that the female participants wanted their body to resemble the avatar created form their real body measures, when perceiving it from a third person perspective (median = 5). The results about attractiveness show that the Ideal body and Real body were evaluated as significantly more attractive when perceived in third person perspective. Logistic regression shows that the Real Body and Ideal Body were rated as significantly more attractive than the Body Image (*p* = 0.002) and that there was a borderline effect of Perspective (*p* = 0.052). These results show that seeing their real body shape in 3PP led female participants to evaluate it more positively (see Discussion).

In the next section we show the results obtained for the same questionnaire on subjective evaluation of the avatars for male participants.

#### Results for male participants

**Figure 13:**
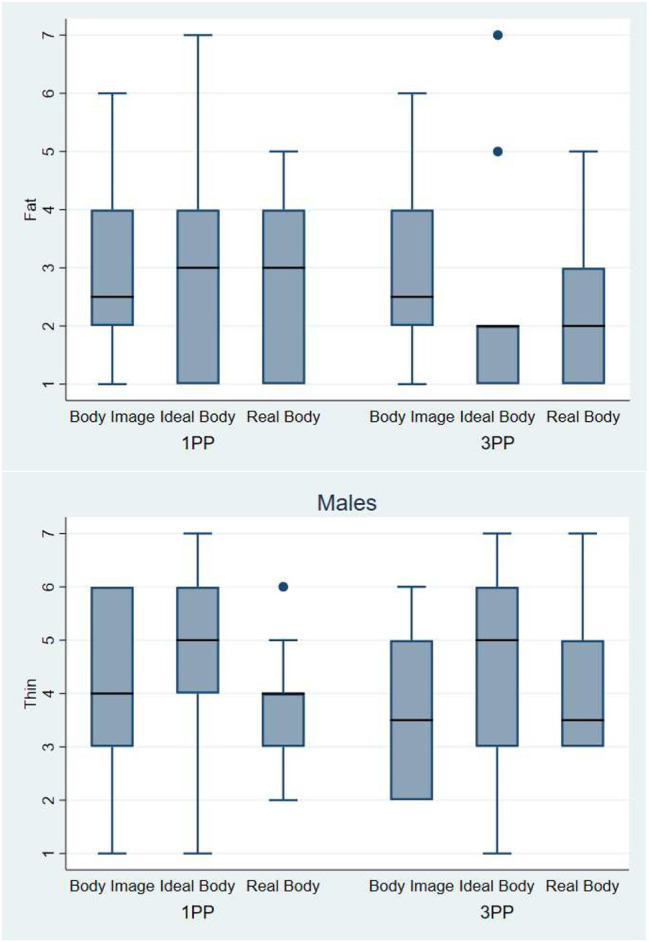
On the top, Box plot presenting the scores obtained during the body evaluation phase. Answers to the question: *How fat do you think this body is*?—Below, Box plot presenting the scores obtained during the body evaluation phase. Answers to the question: *How thin do you think this body is*? The horizontal thick lines indicate the value of the medians, the boxes are the interquartile ranges (IQR), the whiskers extend from max (median - 1.5*IQR, smallest value) to min (median + 1.5*IQR, largest value). Outliers are shown individually as separated dots.

In males the effect of evaluating the Body Image as fatter than the other virtual bodies was not observed. All the medians have similar values (between 2 and 3) for all the avatars in both conditions (1PP and 3PP) (Figure 13).

**Figure 14:**
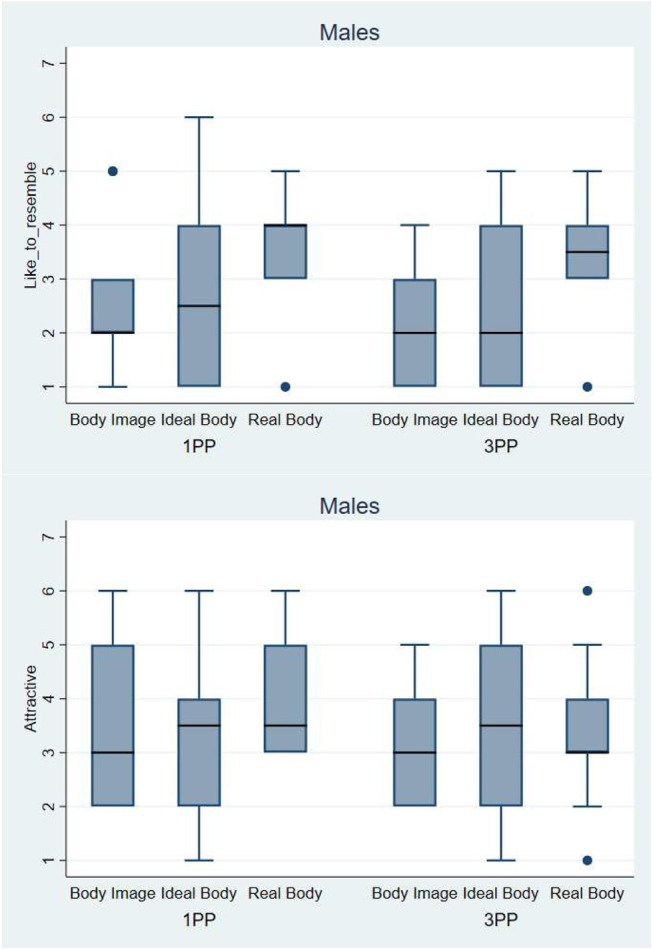
On the top, Box plot presenting the scores obtained during the body evaluation phase. Answers to the question: *How much would you like your body to resemble this one?*—Below, Box plot presenting the scores obtained during the body evaluation phase. Answers to the question: *How attractive does this body look to you?-* The horizontal thick lines indicate the value of the medians, the boxes are the interquartile ranges (IQR), the whiskers extend from max (median - 1.5*IQR, smallest value) to min (median + 1.5*IQR, largest value). Outliers are shown individually as separated dots.

One effect observed in female participants was also observed in males: to the question “How much would you like your body to resemble this one?”, the Real Body was the one that was preferred (median between 3 and 4). But there was no effect of perspective. In males the evaluation of the attractiveness was similar for all the virtual bodies in both 1PP and 3PP conditions. All the medians are between 3 and 4 (Figure 14).

**Figure 15:**
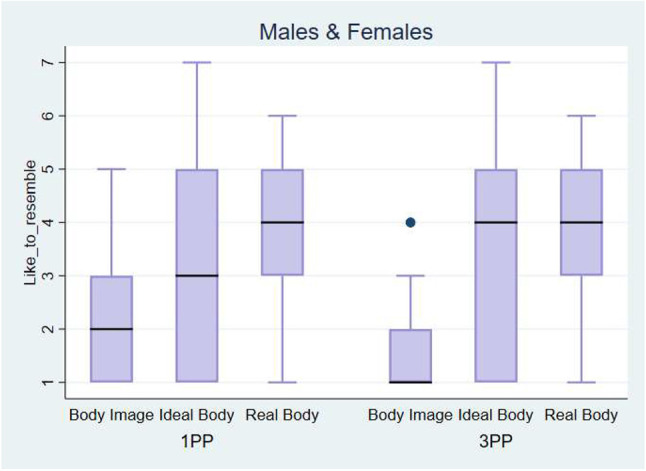
Box plot presenting the scores obtained during the evaluation phase. Answers to the question: *How much would you like your body to resemble this one*. The horizontal thick lines indicate the value of the medians, the boxes are the interquartile ranges (IQR). Outliers are shown individually as separated dots.

We combined the results of all participants, males and females for the question “*How much would you like your body to resemble this one?”* (Figure 15). It is interesting to note that participants wanted their body to resemble the avatar that was constructed from their real body measures. When the Real Body avatar was seen in a third person perspective, that effect was higher, but only in females. This means that our hypothesis was partially true, we can give a new perspective on people's real body, leading them to wanting to have the body they already have when seeing it in 3PP.

### Body Identification

In the last phase of this experiment, participants could view the three avatars in 3PP, one next to the other, and choose which avatar best represented their real body, and which body shape they would like to have (see Table S6). It is interesting to note that no female participant chose their Body Image as the Body they would like to have. A majority of the male participants recognized the correct avatar corresponding to their real body (see Table S7). Overall, participants tended to choose their real body as the one they would like to have (see Table S8). During the post experiment feedback, we observed some participants being really surprised and satisfied to see that the body shape they preferred was actually their real body shape.

## Discussion

As we saw in the introduction, there is an internal representation of one's body based on stored visual information from visual perception of oneself and of others. In this experiment, we explored the implicit mental representation of one's body shape. We created a virtual representation of the internal image participants had of their own body shape, the stored model of their body characteristics generated from all the memories relative to the visual perception of themselves. We use the term “body image” to refer to the internal representation of the visual aspect of one's body shape. As has been observed in previous studies, we expected this internal model to be inaccurate, distorted, and showing an overestimation of body part widths (Longo and Haggard, 2010, 2012; Thaler et al., 2018b). We also expected this model to be vulnerable to pre-existing beliefs about the self and cognitive-affective factors (Garner and Garfinkel, 1980; Mussap and Salton, 2006).

Our results suggest that embodiment and change in perspective affected the evaluation of the attractiveness of a virtual body. When the same virtual body was perceived from a third person perspective, it was evaluated as more attractive than when it was perceived in first person perspective. We hypothesize that prior beliefs about the self and affective factors associated to self-others comparison could be responsible for this effect.

Previous studies have explored the effect of visual perspective on body perception. In Mölbert et al. (2018) the experimenters created virtual bodies that had the participant's body shape and weight using a 3D scanner. The texture applied on these bodies was changed in order to modify the appearance of the avatar while keeping the same body shape. Their results showed that both the desired body and estimated body were slightly thinner than the real body of the participants. In order to generate the estimated body, participants saw different versions of their body and had to decide which version was their real body (depictive method). With our method (metric) we measured a different level of body representation (internal model, divided into body parts) but the desired body (ideal body) was also thinner than the estimated body (body image).

In our study, we observed a negative bias when the virtual body was perceived in 1PP: the negative pre-existing beliefs about the self directly affected the interpretation of the visual stimulus associated to self-perception (the virtual body). Changing the perspective, the virtual body is no longer associated to the self, but perceived as the body of someone else, therefore the prior beliefs applied to the visual stimulus are modified, the interpretation of this visual stimulus is changed, and the evaluation of the virtual body is modified. Recent studies show that visual perspective in virtual reality has a direct impact on body perception, it has been observed that males and females underestimated their body weight on a virtual body viewed from a third-person perspective (Pujades et al., 2019; Thaler et al., 2019).

In our experiment we observed that female participants wanted their body to resemble the avatar that was recreated from their real body shape. When this real body was seen in a third person perspective, that effect was higher. In the “body identification” task, more than half of the participants chose their “Real body” as the one they would like to have. At the end of the procedure, when feedback identifying the virtual bodies was given to the participants, many of them were greatly surprised to know that the body they preferred was actually their real one. In females we observed that body dissatisfaction scores were decreased after the experimental procedure. We believe that seeing their real body from a third person perspective helped participants to decrease dissatisfaction about their body shape.

We can argue that this negative bias toward the self might be produced by the comparison between self and others. Our female participants seemed to apply a systematic negative evaluation to the virtual body when they perceived it as their own body (1PP), but not when the virtual body was presented as someone else's body (3PP). In the last phase (Body identification), participants did not know that the body they were choosing as “the one they wanted to have” was actually their own body. We suppose that the negative bias about the self was modified because the body was seen in third person perspective. We know that negative prior beliefs about the self are higher in patients with mental disorders (Wong, 2008), and when it comes to prior beliefs about body shape, negative bias is particularly prominent in patients with eating disorders (Williamson, 1996). We believe that seeing their real body in 3PP could be therapeutic for those patients if we obtain the same effect as in this study with healthy participants. This procedure could help them to understand that the internal representation they have of their own body is highly inaccurate, and that the ideal body representation they aim to reach is incompatible with the internal equilibrium and safety of their body. It has been shown that patients with eating disorders pay more attention to the visual stimuli related to their bodily self than to their interoceptive information (Eshkevari et al., 2012, 2013). We believe that this procedure could help them understand that their body perception is inaccurate and affected by the negative prior beliefs they have about themselves. Based on the results obtained in this first study, we believe that, by perceiving their body in 3PP, patients could obtain a new and unbiased perception of their own body (as if it were the body of someone else). This new perception could re-orientate their attention to the real features of their body shape in a more accurate and objective way.

We surmise that the effect observed with this small sample of healthy participants predicts that, in patients with eating disorders, the effect might be stronger. In the case of patients, body size overestimation is increased and body representation may drastically differ from the real body (Gardner, 1996; Gardner and Brown, 2014). We believe that our method could be an effective way to update body representation in patients with a distorted body image (Keizer et al., 2016).

We observed that, according to their gender, the ideal bodies imagined by our participants all had similar features to one another (e.g., male bodies with large biceps and female bodies with narrow waists). Our female participants showed a general tendency toward an overestimation of their own body and an underestimation of the bodily measures for their ideal body shape (see Figure 9). As stated above, an overestimation of one's body parts is consistent with previous findings and can be due to the use of a metric method (Longo and Haggard, 2012; Thaler et al., 2018b). One hypothesis explaining this common overestimation is that there is a margin of error in the representation of the body for navigating safely through space (Warren and Whang, 1987). More interestingly in the case of our results, previous findings suggested that estimation of emotionally salient body regions could be affected by socio-affective factors (Garner and Garfinkel, 1980; Ben-Tovim and Walker, 1990). We observe in our data that the overestimated body parts were indeed the waist, hips and chest for the females. It is the same body parts that were highly distorted in the representation of the ideal body shape.

In the study conducted by Thaler et al. (2019), a depictive method was used for participants to estimate their body measures inside a virtual environment, and create an avatar representing their body shape. It is important to note that in that study, mirror was considered as 3PP view and only look down was considered as 1PP. Both males and females underestimated their weight by 10–20%. In another study by Mölbert et al. (2018) the estimated body was also slightly thinner than the real body. In our case, on the contrary, there was an overestimation of the body for both males and females. This overestimation was more important in females and specific to body parts associated to the socio-cultural ideal body shape. We argue that this difference in the results obtained in both studies cited above and our experiment, comes from the use of a metric method, and maybe from socio-affective differences between the Spanish population and the German population. It would be interesting to investigate this cultural aspect further. In the literature, cultural differences affected body size estimation, but across different ethnic groups. For instance, African American women perceived their body size as smaller than White women with equivalent BMIs (Kronenfeld et al., 2010). Differences in body satisfaction according to size were also observed in a multi-ethnic group showing that South Asian males were also more likely to overestimate their body size than the other adolescents (Simeon et al., 2003).

The results we obtained for the estimation of the ideal body for female participants are consistent with the results obtained by Gardner and Moncrieff (1988). In their study, the average ideal body in females showed a very important chest circumference and a very small waist circumference. It is interesting to note that the “ideal body shape” of our participants corresponds to the “hourglass figure”, and that their average “body image” corresponds to the “rectangle figure” reported in Simmons et al. (2004). It seems that our female participants idealized the “hourglass figure” and assumed that their body shape was corresponding to the “rectangle figure.” The ideal female body reported in our experiment, corresponds to the ideal female body obtained in the study by Crossley et al. (2012), and is impossible to be found in reality. The virtual body representing the “ideal body shape” of our female participants presented the same body features as the “ideal figure” reported in that study: a chest circumference substantially larger than the one attributable to the overall thinness of the other parts of that body (Crossley et al., 2012).

It is particularly relevant to note here that a person's internal representation of their “idealized body” is highly defined by prior beliefs coming from the culture in which they have grown up (Thompson and Stice, 2001). As explained above, the social environment shapes the prior beliefs of individuals and influences their body perception, the cultural aesthetic ideal is internalized and affects body image (Candidi and Aglioti, 2015). In our results, consistent with previous findings as described above, the “ideal body shape” shows an increased circumference of the chest that is incompatible with the thinness of the hips and waist, therefore it is not a realistic body shape. The desire to reach this “ideal body shape” is motivated by the will to obtain an increased social acceptance. This social motivation enters in conflict with maintaining the internal equilibrium (homeostasis) of the body. This phenomenon can be observed in many females at different degrees of importance and is an example of detrimental interaction between the individuals and their social environment. In the case of eating disorders, it can have neurochemical consequences threatening the equilibrium of the body (Bergh and Södersten, 1996; Södersten et al., 2014, 2016). The self-starvation behavior is associated to the reward expectancy of reaching an ideal social criterion, and food intake is not a rewarding behavior anymore (Hohlstein et al., 1998; Gretha et al., 2010). In females with eating disorders, this can lead to severe neurobiological consequences, neurochemical disorders and even to death. Several networks of motivations can co-exist and enter in conflict (Menon, 2015). In the case of eating disorders, it seems that “social desirability” co-exists with the primary program of “food intake”, aimed at survival and preservation of homeostasis within the body.

We know that environmentally driven processes select the synaptic connections that are most effectively entrained to environmental information (Schore, 2001). In the case of anorexia nervosa, we can argue that the behaviors associated with reaching the “ideal body shape” are highly adapted to socio-environmental “requirements”, but enter in conflict with the most basic behaviors of “food seeking” and “food intake” (Garner and Garfinkel, 1980). One of the motivational networks becomes salient and leads to the onset of pathological behaviors. Patterns learnt through interaction with the social environment can lead to un-adaptive beliefs and pathological behaviors. In this experiment, we showed the existence of a pattern defining a cultural “ideal body shape”, by reconstructing the ideal avatar imagined by our participants. We have seen that it corresponds to the “ideal body shape” observed in other studies (Simmons et al., 2004; Crossley et al., 2012). We postulate that, comparing one's body image with one's real body and one's ideal body, would be particularly relevant for patients with eating disorders given their sensitivity to the cultural ideal body. We believe that our method could help them in updating the biased representation they have of their own body, by seeing it from a third person perspective.

## Conclusions

Our results show that the avatars created, based on the ideal measures given by our participants, all had similar physical attributes, showing the prominence of an “ideal body shape” salient in the cultural environment. We also showed that the internal representation that people create of their own body is highly inaccurate. By showing their real body to our female participants from a third person perspective, we made it appear more attractive to them. We believe that this method can be particularly efficient for increasing body satisfaction in patients with eating disorders. One further development of this method would be to use it as diagnostic tool for body perception disorders.

## Data Availability Statement

The datasets generated for this study are available on request to the corresponding author.

## Ethics Statement

The studies involving human participants were reviewed and approved by Comissio Bioetica Universitat de Barcelona. The patients/participants provided their written informed consent to participate in this study.

## Author Contributions

SN and MS designed the overall concept. MS obtained the funding. SN, AB, and MS designed the experiment. SN determined the psychological assessment indicators and wrote the paper with the help of all the other authors. AB and XN designed and implemented the computer program. AB designed and carried out the creation of the avatars. SN and AB carried out the experiment and prepared the data. MS and SN analyzed the data. All authors checked this and contributed to the final version.

### Conflict of Interest

The authors declare that the research was conducted in the absence of any commercial or financial relationships that could be construed as a potential conflict of interest.

## References

[B1] Ben-Tovim D. I. Walker M. K. (1990). Effect of a mirror on body-size estimation. Percept. Mot. Skills 71, 1151–1154. 10.2466/pms.1990.71.3f.1151 2087369

[B2] Bergh C. Södersten P. (1996). Anorexia nervosa, self-starvation and the reward of stress. Nat. Med. 2, 21–22. 10.1038/nm0196-21 8564826

[B3] Bermúdez J. L. Marcel A. J. Eilan N. (Eds.). (1995). The Body and the Self. Washington, DC: The MIT Press.

[B4] Bourdin P. Barberia I. Oliva R. Slater M. (2017). A virtual out-of-body experience reduces fear of death. PLoS ONE 12:e0169343. 10.1371/journal.pone.0169343 28068368 PMC5221792

[B5] Calvo-Merino B. Glaser D. E. Grèzes J. Passingham R. E. Haggard P. (2005). Action observation and acquired motor skills: an FMRI study with expert dancers. Cereb. Cortex 15, 1243–1249. 10.1093/cercor/bhi007 15616133

[B6] Candidi M. Aglioti S. M. (2015). Visual and sensorimotor contributions to the esthetic appraisal of body form, motion, and emotion. Eur. Psychol. 20, 16–26. 10.1027/1016-9040/a000221

[B7] Cattarin J. A. Thompson J. K. Thomas C. Williams R. (2000). Body image, mood, and televised images of attractiveness: the role of social comparison. J. Soc. Clin. Psychol. 19, 220–239. 10.1521/jscp.2000.19.2.220

[B8] Cooper P. J. Cooper M. J. Cooper Z. Fairburn C. G. (1987). The development and validation of the body shape questionnaire. Int. J. Eat. Disord. 6, 485–494. 10.1002/1098-108X(198707)6:4<485::AID-EAT2260060405>3.0.CO;2-O

[B9] Crossley K. L. Cornelissen P. L. Tovée M. J. (2012). What is an attractive body? using an interactive 3D program to create the ideal body for you and your partner. PLoS ONE 7:e50601. 10.1371/journal.pone.0050601 23209791 PMC3510069

[B10] de Vignemont F. (2010). Body schema and body image–pros and cons. Neuropsychologia 48, 669–680 10.1016/j.neuropsychologia.2009.09.022 19786038

[B11] de Vignemont F. (2011). Embodiment, ownership and disownership. Conscious. Cogn. 20, 82–93. 10.1016/j.concog.2010.09.004 20943417

[B12] Decety J. (1996). The neurophysiological basis of motor imagery. Behav. Brain Res. 77, 45–52. 10.1016/0166-4328(95)00225-1 8762158

[B13] Eshkevari E. Rieger E. Longo M. R. Haggard P. Treasure J. (2012). Increased plasticity of the bodily self in eating disorders. Psychol. Med. 42, 819–828. 10.1017/S0033291711002091 22017964

[B14] Eshkevari E. Rieger E. Longo M. R. Haggard P. Treasure J. (2013). Persistent body image disturbance following recovery from eating disorders. Int. J. Eat. Disord. 47, 400–409. 10.1002/eat.22219 24243423

[B15] Gallagher I. (2000). Philosophical conceptions of the self: implications for cognitive science. Trends in Cogn. Sci. 4, 14–21. 10.1016/S1364-6613(99)01417-5 10637618

[B16] Gallagher S. (1986). Body image and body schema: conceptual clarification. J. Mind Behav. 7, 541–554.

[B17] Gallagher S. (2005). How the Body Shapes the Mind, Vol. 20 Leonardo: Oxford University Press.

[B18] García-García E. Vázquez-Velázquez V. López-Alvarenga J. C. Arcila-Martínez D. (2003). Validez interna y utilidad diagnóstica del eating disorder inventory, en mujeres Mexicanas. Salud Pública de México 45. 10.1590/S0036-36342003000300010 12870422

[B19] Gardner R. M. (1996). The role of sensory and nonsensory factors in body size estimations of eating disorder subjects. Clin. Psychol. 52, 3–15. 10.1002/(SICI)1097-4679(199601)52:1<3::AID-JCLP1>3.0.CO;2-X 8682909

[B20] Gardner R. M. Brown D. L. (2014). Body size estimation in anorexia nervosa: a brief review of findings from 2003 through 2013. Psychiatry Res. 219, 407–410. 10.1016/j.psychres.2014.06.029 25023364

[B21] Gardner R. M. Moncrieff C. (1988). Body image distortion in anorexics as a non-sensory phenomenon: a signal detection approach. J. Clin. Psychol. 44, 101–107. 10.1002/1097-4679(198803)44:2<101::aid-jclp2270440203>3.0.co;2-u 3360922

[B22] Garner D. M. Garfinkel P. E. (1980). Socio-cultural factors in the development of anorexia nervosa. Psychol. Med. 10, 647–656. 10.1017/S0033291700054945 7208724

[B23] Garner D. M. Garfinkel P. E. (1981). *Body image in anorexia nervosa:* Measurement, theory and clinical implications. Int. J. Psychiatry Med. 11, 263–84. 10.2190/r55q-2u6t-lam7-rqr7 7309395

[B24] Garner D. M. Olmstead M. P. Polivy J. (1983). Development and validation of a multidimensional eating disorder inventory for anorexia nervosa and bulimia. Int. J. Eat. Disord. 2, 15–34. 10.1002/1098-108X(198321)2:2<15::AID-EAT2260020203>3.0.CO;2-6

[B25] Gretha J. Scheurink A. J. W. Boersma G. J. Nergårdh R. Södersten P. (2010). Neurobiology of hyperactivity and reward publisher's PDF, also known as version of record publication date : physiology & behavior neurobiology of hyperactivity and reward : agreeable restlessness in Anorexia Nervosa. Physiol. Behav. 100, 490–495. 10.1016/j.physbeh.2010.03.016 20361989

[B26] Hohlstein L. A. Smith G. T. Atlas J. G. (1998). An application of expectancy theory to eating disorders: development and validation of measures of eating and dieting expectancies. Psychol. Assess. 10, 49–58. 10.1037/1040-3590.10.1.49

[B27] Kammers M. P. M. Longo M. R. Tsakiris M. Chris Dijkerman H. Haggard P. (2009). Specificity and coherence of body representations. Perception 38, 1804–1820. 10.1068/p6389 20192130

[B28] Keizer A. Van Elburg A. Helms R. Dijkerman H. C. (2016). A virtual reality full body illusion improves body image disturbance in anorexia nervosa. PLoS ONE 11:e0163921. 10.1371/journal.pone.0163921 27711234 PMC5053411

[B29] Kronenfeld L. W. Reba-Harrelson L. Von Holle A. Reyes M. L. Bulik C. M. (2010). Ethnic and racial differences in body size perception and satisfaction. Body Image 7, 131–136. 10.1016/j.bodyim.2009.11.002 20096656 PMC3593344

[B30] Longo M. R. Haggard P. (2010). An implicit body representation underlying human position sense. Proc. Natl. Acad. Sci. U.S.A. 107, 11727–11732. 10.1073/pnas.1003483107 20547858 PMC2900654

[B31] Longo M. R. Haggard P. (2012). Implicit body representations and the conscious body image. Acta Psychol. 141, 164–168. 10.1016/j.actpsy.2012.07.015 22964057

[B32] Longo M. R. Schüür F. Kammers M. P. M. Tsakiris M. Haggard P. (2009). Self awareness and the body image. Acta Psychol. 20, 233–252. 10.1016/j.actpsy.2009.02.003 19286139

[B33] Menon V. (2015). Salience Network. in Brain Mapping: An Encyclopedic Reference, Vol. 2 Academic Press; Elsevier. 10.1016/B978-0-12-397025-1.00052-X

[B34] Metzinger T. (2014). First-order embodiment, second-order embodiment, third-order embodiment, in The Routledge Handbook of Embodied Cognition, ed Shapiro L. (Abbingdon; Oxon: Routledge/Taylor & Francis Group), 272–286.

[B35] Mölbert S. C. Thaler A. Mohler B. J. Streuber S. Romero J. Black M. J.. (2018). Assessing body image in anorexia nervosa using biometric self-avatars in virtual reality: attitudinal components rather than visual body size estimation are distorted. Psychol. Med. 48, 642–653. 10.1017/S0033291717002008 28745268 PMC5964466

[B36] Mussap A. J. Salton N. (2006). A rubber-hand illusion reveals a relationship between perceptual body image and unhealthy body change. J. Health Psychol. 11, 627–639. 10.1177/1359105306065022 16769741

[B37] Myers P. N. Biocca F. A. Caralina N. (1992). The elastic body image: the effect of television advertising and programming on body image distortions in young women. J. Commun. 42, 108–133. 10.1111/j.1460-2466.1992.tb00802.x

[B38] Naito E. Kochiyama T. Kitada R. Nakamura S. Matsumura M. Yonekura Y.. (2002). Internally simulated movement sensations during motor imagery activate cortical motor areas and the cerebellum. J. Neurosci. 22, 3683–3691. 10.1523/JNEUROSCI.22-09-03683.2002 11978844 PMC6758350

[B39] Neisser U. (2008). Five kinds of self - knowledge. Philos. Psychol. 1, 35–59. 10.1080/09515088808572924

[B40] Nimcharoen C. Zollmann S. Collins J. Regenbrecht H. (2019). Is that me? - embodiment and body perception with an augmented reality mirror, in Adjunct Proceedings - 2018 IEEE International Symposium on Mixed and Augmented Reality, ISMAR-Adjunct 2018 (New York, NY). 10.1109/ISMAR-Adjunct.2018.00057

[B41] Perez-Marcos D. Martini M. Fuentes C. T. Bellido Rivas A. I. Haggard P. Sanchez-Vives M. V. (2018). Selective distortion of body image by asynchronous visuotactile stimulation. Body Image 24, 55–61. 10.1016/j.bodyim.2017.11.002 29268137

[B42] Petkova V. I. Ehrsson H. H. (2008). If I were you: perceptual illusion of body swapping. PLoS ONE 3:e3832. 10.1371/journal.pone.0003832 19050755 PMC2585011

[B43] Piryankova I. V. Stefanucci J. K. Romero J. De La Rosa S. Black M. J. Mohler B. J. (2014a). Can i recognize my body's weight? The influence of shape and texture on the perception of self. ACM Trans. Appl. Percept. 11:3 10.1145/2641568

[B44] Piryankova I. V. Wong H. Y. Linkenauger S. A. Stinson C. Longo M. R. Bülthoff H. H.. (2014b). Owning an overweight or underweight body: Distinguishing the physical, experienced and virtual body. PLoS ONE. 9:e103428. 10.1371/journal.pone.0103428 25083784 PMC4118886

[B45] Pujades S. Mohler B. Thaler A. Tesch J. Mahmood N. Hesse N.. (2019). The virtual caliper: rapid creation of metrically accurate avatars from 3D measurements. IEEE Trans. Vis. Comput. Graph. 25, 1887–1897. 10.1109/TVCG.2019.2898748 30794512

[B46] Raich R. M. Mora M. Soler A. Avila C. Clos I. Zapater L. (1996). Adaptación de un instrumento de evaluación de la insatisfacción corporal. [Adaptation of a body dissatisfaction assessment instrument]. Clínica y Salud. 7, 51–66.

[B47] Schore A. (2001). Effecs of a secure attachment relationship on right brain development, affect regulation, and infant mental health. Infant Ment. Health J. 22, 7–66. 10.1002/1097-0355(200101/04)22:1<7::AID-IMHJ2>3.0.CO;2-N

[B48] Simeon D. T. Rattan R. D. Panchoo K. Kungeesingh K. V. Ali A. C. Abdool P. S. (2003). Body image of adolescents in a multi-ethnic Caribbean population. Eur. J. Clin. Nutr. 57, 157–162. 10.1038/sj.ejcn.1601515 12548311

[B49] Simmons K. Istook C. L. Devarajan P. (2004). Female figure identification technogue (FFIT) for apparel part II: development of shape sorting software. J. Textile Apparel, Technol. Manag. 4 Available online at: https://www.researchgate.net/publication/238103641

[B50] Södersten P. Bergh C. Leon M. Zandian M. (2016). Dopamine and anorexia nervosa. Neurosci. Biobehav. Rev. 60, 26–30. 10.1016/j.neubiorev.2015.11.003 26608248

[B51] Södersten P. Bergh C. Zandian M. Ioakimidis I. (2014). Homeostasis in anorexia nervosa. Front. Neurosci. 8:234. 10.3389/fnins.2014.00234 25147496 PMC4123620

[B52] Thaler A. Bülthoff I. Pujades S. Black M. Mohler B. (2018a). Is body size estimation viewpoint invariant? J. Vis. 18:165 10.1167/18.10.165

[B53] Thaler A. Geuss M. Molbert S. Giel K. Streuber S. Black M. (2016). Investigating the influence of personal BMI on own body size perception in females using self-avatars. J. Vis. 16:400 10.1167/16.12.1400

[B54] Thaler A. Geuss M. Stefanucci J. Mölbert S. Giel K. Black M. (2017). Perception of others' body sizes is predicted by own body size. J. Vis. 17:843 10.1167/17.10.843

[B55] Thaler A. Geuss M. N. Mohler B. J. (2018b). The role of visual information in body size estimation. I Perception 9:2041669518796853. 10.1177/2041669518796853 30202510 PMC6128079

[B56] Thaler A. Geuss M. N. Mölbert S. C. Giel K. E. Streuber S. Romero J.. (2018c). Body size estimation of self and others in females varying in BMI. PLoS ONE 13:e0192152. 10.1371/journal.pone.0192152 29425218 PMC5806871

[B57] Thaler A. Pujades S. Stefanucci J. K. Creem-Regehr S. H. Tesch J. Black M. J. (2019). The influence of visual perspective on body size estimation in immersive virtual *Reality,* in ACM Symposium on Applied Perception (New York, NY: ACM publication). 10.1145/3343036.3343134

[B58] Thompson J. K. Stice E. (2001). Thin-ideal internalization: mounting evidence for a new risk factor for body-image disturbance and eating pathology. Current. Direct. Psychol. Sci. 10, 181–183. 10.1111/1467-8721.00144

[B59] Tsakiris M. (2016). The multisensory basis of the self: from body to identity to others. Q. J. Exp. Psychol. 70, 597–609. 10.1080/17470218.2016.1181768 27100132 PMC5214748

[B60] Warren W. H. Whang S. (1987). Visual guidance of walking through apertures: body-scaled information for affordances. J. Exp. Psychol. Hum. Percept. Perform. 13, 371–383. 10.1037/0096-1523.13.3.371 2958586

[B61] Williamson D. A. (1996). Body image disturbance in eating disorders: a form of cognitive bias? Eat. Disord. 4, 47–58. 10.1080/10640269608250075

[B62] Wong S. S. (2008). The relations of cognitive triad, dysfunctional attitudes, automatic thoughts, and irrational beliefs with test anxiety. Curr. Psychol. 27, 177–199. 10.1007/s12144-008-9033-y

