# Supplementary material for: Which Body Would You Like to Have? The Impact of Embodied Perspective on Body Perception and Body Evaluation in Immersive Virtual Reality
Source: Front Robot AI. 2020 Mar 18;7:31. doi: 10.3389/frobt.2020.00031 (PMC7805750; doi:10.3389/frobt.2020.00031)
Supplement: Supplementary file 1 [file Data_Sheet_1.pdf]

Which body would you like to have? The impact of embodied perspective on body perception and body evaluation in immersive virtual reality

Authors: Solène Neyret, Anna I. Bellido Rivas, Xavi Navarro, Mel Slater

SUPPLEMENTARY MATERIAL:

PARTICIPANTS

There was no significant difference in the age of the participants, one participant was a bit older than the average (38 years old) but this did not affect the results obtained.

Table S1: Age characteristics of the participants.

	Age (mean ± SD)	Age (Min)	Age (Max)
Total (n=19)	24.8 ± 5.64	18	38
Females (n=9)	23.9 ± 6.79	18	38
Males (n=10)	25.6 ± 4.59	19	32

The BMI for both genders were distributed in the range of healthy weight, there was one female underweight and one male overweight. The BMI is calculated as follows: Weight/ (Height × Height).

Table S2: BMI (body mass index) characteristics of the participants. The scale is as follows, 16.0 to 18.5: underweight, 18.5 to 25: healthy weight, 25 to 30: overweight, 30 to 35: Moderately obese

	BMI (mean ± SD)	BMI (Min)	BMI (Max)
Total (n=19)	23.6 ± 3.42	17.75	33.33
Females (n=9)	22.8 ± 3.31	17.75	27.25
Males (n=10)	24.3 ± 3.54	20.30	33.33

CLINICAL QUESTIONNAIRES

BSQ-34

Table S3: Scores obtained in the body shape questionnaire (BSQ), the scale is as follows, <81: no concern with shape, 81-110: low concern with shape, 111-140: moderate concern with shape, >140: concern with shape.

	BSQ_pre (mean ± SE)	BSQ_post (mean ± SE)
Females (n=9)	80.55 ± 4.26	80.55 ± 5.74
Males (n=10)	53.6 ± 4.84	58.7 ± 4.53

EDI-2

Table S4: Scores of the Drive for thinness (DT) subscale of the EDI-2 questionnaire. The scale is as follows, 0: percentile 32, 2: percentile 59, 4: percentile 78, 17: percentile 99

	DT_pre (mean ± SE)	DT_post (mean ± SE)
Females (n=9)	3.44 ± 0.83	2.55 ± 0.75
Males (n=10)	0.4 ± 0.22	0.6 ± 0.43

Table S5: Scores of the Body dissatisfaction (BD) subscale of the EDI-2 questionnaire. The scale is the following, 0: percentile 26, 3: percentile 56, 6: percentile 75, 24: percentile 99

	BD_pre (mean ± SE)	BD_post (mean ± SE)
Females (n=9)	7.11 ± 1.83	3.89 ± 1.49
Males (n=10)	1.2 ± 0.76	2.3 ± 1.00

BODY IDENTIFICATION

Table S6: Final evaluation with the three avatars in front of the participant, frequency of choice for Females

Females	Which body do you think is your real body?	Which body do you think is your ideal body?	Which body would you like to have?
Body Image	3	1	**0**
Ideal Body	1	5	4
Real Body	5	3	5

Table S7: Final evaluation with the three avatars in front of the participant, frequency of choice for Males

Males	Which body do you think is your real body?	Which body do you think is your ideal body?	Which body would you like to have?
Body Image	3	4	**5**
Ideal Body	0	4	1
Real Body	**7**	2	4

Table S8: Final evaluation with the three avatars in front of the participant, frequency of choice for Males and Females

Total	Which body do you think is your real body?	Which body do you think is your ideal body?	Which body would you like to have?
Body Image	6	5	5
Ideal Body	1	**9**	5
Real Body	**12**	5	**9**
